# Effects of a buried magnetic field on cranial bone reconstruction in rats

**DOI:** 10.1590/1678-775720150336

**Published:** 2016

**Authors:** Maíra Cavallet de ABREU, Deise PONZONI, Renan LANGIE, Felipe Ernesto ARTUZI, Edela PURICELLI

**Affiliations:** Universidade Federal do Rio Grande do Sul, Faculdade de Odontologia, Departamento de Cirurgia Oral e Maxilofacial, Hospital de Clínicas de Porto Alegre, Porto Alegre, RS, Brasil.

**Keywords:** Maxillofacial surgery, Magnetic field therapy, Bone substitutes

## Abstract

The understanding of bone repair phenomena is a fundamental part of dentistry and maxillofacial surgery.

**Objective:**

The present study aimed to evaluate the influence of buried magnetic field stimulation on bone repair in rat calvaria after reconstruction with autogenous bone grafts, synthetic powdered hydroxyapatite, or allogeneic cartilage grafts, with or without exposure to magnetic stimulation.

**Material and Methods:**

Ninety male Wistar rats were divided into 18 groups of five animals each. Critical bone defects were created in the rats’ calvaria and immediately reconstructed with autogenous bone, powdered synthetic hydroxyapatite or allogeneic cartilage. Magnetic implants were also placed in half the animals. Rats were euthanized for analysis at 15, 30, and 60 postoperative days. Histomorphometric analyses of the quantity of bone repair were performed at all times.

**Results:**

These analyses showed significant group by postoperative time interactions (p=0.008). Among the rats subjected to autogenous bone reconstruction, those exposed to magnetic stimulation had higher bone fill percentages than those without magnetic implants. Results also showed that the quality of bone repair remained higher in the former group as compared to the latter at 60 postoperative days.

**Conclusions:**

After 60 postoperative days, bone repair was greater in the group treated with autogenous bone grafts and exposed to a magnetic field, and bone repair was most pronounced in animals treated with autogenous bone grafts, followed by those treated with powdered synthetic hydroxyapatite and allogeneic cartilage grafts.

## INTRODUCTION

The successful reconstruction of oral and maxillofacial bone defects has been recently enabled by advances in the understanding of bone physiology along with improvements in surgical techniques. Such improvements have allowed the development of techniques that promote biological repair, reestablishing the function of damaged tissues. Many biological, chemical, and physical stimuli have been found to have a positive influence on bone growth, repair, and remodeling. An example of such a stimulus is magnetic field stimulation, which has been found to have positive effects on tissue, cellular, and molecular processes^1^ ^,^ ^8^ ^,^ ^20^ ^,^ ^21^.

Several studies have been performed in order to assess the effects of static magnetic fields on different types of tissues^27^. Magnetic fields applied through the skin may activate iron atoms in hemoglobin, influencing oxygen transport, and stimulate osteogenesis by the activation of osteoblasts and by leading to an increase in blood flow to bone^6^ ^,^ ^20^ ^,^ ^21^. Additionally, results suggest that magnetic fields may increase the concentration of growth factors, accelerating the bone repair process^1^. Magnet therapy can be a treatment option, since static magnetic field have a positive influence on bone metabolism^4^.

The results of studies on the effects of magnetic stimulation on tissue repair are still controversial. There has been some favorable evidence of the effects of static magnetic fields generated by permanent magnets or by magnetized materials. Many studies have shown, for instance, that static magnetic fields can accelerate the repair of bones or tissues^6^ ^,^ ^20^ ^,^ ^21^.

The present study aimed to evaluate the influence of buried magnetic field stimulation on bone repair in rat calvaria after reconstruction with autogenous bone grafts, powdered synthetic hydroxyapatite or allogeneic cartilage, using bone histomorphometric analysis.

## MATERIAL AND METHODS

The use of animals in the present study conformed to the State Code for Animal Protection and was in accordance with Normative Resolution 04/97 of the Research Ethics Committee of our institution (GPPG/HCPA), which reviewed and approved the present project (Project no. 10-0307). All experimental procedures were performed in the Animal Experimentation Unit of the Clinical Hospital of Porto Alegre (UEA-HCPA).

Sample size was estimated using G*Power software (version 3). A sample of 90 animals would have 80% power to detect a difference in bone fill percentage between two independent groups with a type I error rate of 5%.

Ninety male Wistar rats aged between seven and eight weeks with an average weight of 300 g were used in the present study. Block randomization was used to assign animals to one of 18 groups composed of five rats each, which were evaluated after 15, 30, and 60 postoperative days. The influence of buried magnetic fields in bone repair was evaluated by the implantation of two permanent magnets (Pan^®^, São Paulo, SP, Brazil) adjacent to a bone defect created in the calvarium. Each animal was individually subjected to a reconstruction procedure using an autogenous bone graft, a powdered synthetic hydroxyapatite implant (HAP 91^®^, JHS Biomateriais, Sabará, MG, Brazil) or an allogeneic cartilage graft.

In the autogenous bone group, the bone graft collected during the creation of the cranial defect was repositioned for reconstruction. The powdered synthetic hydroxyapatite was absorbable and porous, and was prepared using a sieve with a mesh size of 2 mm. It was directly implanted on the receiver, according to manufacturer’s instructions. Allogeneic cartilage grafts were obtained as described by Vieira, et al.^25^ (1993). A piece of cartilage without perichondrium was first harvested, then washed with sterile saline solution for 15 minutes and preserved in a 70% ethanol solution under refrigeration (2° to 8°C) for 20 days. Particulate cartilage grafts were implanted in the bone defect according to the protocol described by Bercini & Puricelli^3^ (1992).

The magnetic field was generated by two neodymium iron boron magnets implanted adjacent to the bone defect. All magnets used in the study were measured by a gauss meter (Magnet-Physik FH 35, Magnet-Physik Steingroever, Köln, Nordrhein-Westfalen, Germany), and the average intensity of the magnetic field in the central region of the bone defect was 84.3G. Commercially pure titanium discs (Promm^®^ Surgical Materials Industry, Porto Alegre, RS, Brazil) were used in the calvaria of control group rats in order to simulate the presence of magnets.

**Figure 1:**
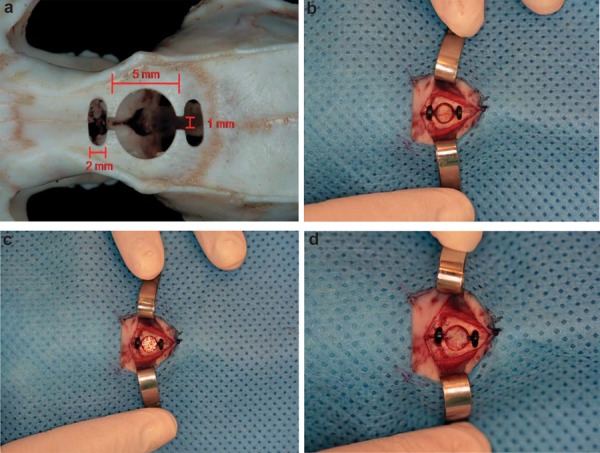
Reconstruction of critical bone defects in rat calvaria using different materials. a) Dimensions of critical bone defects; b) Reconstruction with autogenous bone graft; c) Reconstruction with powdered synthetic hydroxyapatite implant; d) Reconstruction with allogeneic cartilage graft

Strict asepsis was observed during the procedures. The rats were anesthetized by intraperitoneal ketamine hydrochloride (100 mg/kg) and xylazine hydrochloride (10 mg/kg), as well as local bupivacaine (2 mg/kg). A trephine drill (Neodent®, Curitiba, PR, Brazil) was used to create a bicortical defect in the frontal bone, measuring 5 mm in diameter and 1 mm in depth. Two 2 mm osteotomies with 1 mm gaps were created anterior and posterior to the bone defect for the placement of the two magnets. This method was used to facilitate the penetration of the magnetic field in the bone defect. The defect was filled with each of the different materials (Figure 1). During the postoperative period, the rats received food and water, and 5 mg/kg Tramadol for pain relief. Animals were euthanized by decapitation after the previously described postoperative periods.

### Histological preparation

The material was fixed in 10% neutral buffered formalin for 24 hours, after which the pieces were decalcified in a 50% formic acid and 20% sodium citrate solution (1:1). All metallic devices were carefully removed during decalcification. Once the process was complete, a median longitudinal section of the calvarium was taken. The pieces were embedded in paraffin, and 4 µ-histological sections were taken from the central area of the bone defect. Slides were then stained with hematoxylin and eosin (HE) and coded to allow for a blind evaluation.

To ensure correct calibration, ten slides were evaluated twice with an interval of seven days. The values obtained for the two calibration measurements were analyzed using R software (version 2.9.0, R Development Core Team 2010, Auckland, Auckland, Nova Zelândia). The intraclass correlation coefficient (ICC) for the two calibration measurements was 0.98, with a confidence interval (CI) of 0.61 to 0.99.

**Figure 2:**
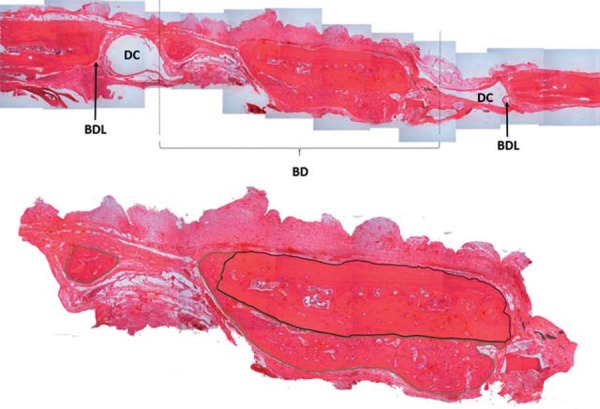
Mosaic-like arrangement of the entire length of the bone defect and central portion of the defect. Superior image: BDL indicates the bone defect limit, DC indicates position of the metal device used (magnet or titanium disk), BD indicates the bone defect area; Inferior image: it shows the image obtained for histomorphometric analysis after exclusion of areas outside the critical bone defect created; the black line delimits the autogenous bone graft and the green line delimits the new bone

The histological field examined included the entire length of the bone defect, and 100X-magnified images of the histological slides were captured by an Olympus^®^ video camera (Model 5, Qcolor Cooler, RTV) coupled to a binocular microscope (Olympus Optical Co.^®^, CX41RF) and a Dell computer (Dimension 5150) running Qcapture^®^ software (version 2.81; Quantitative Imaging Corporation, Inc.; 2005). Photomicrographs were grouped side by side in a mosaic-like arrangement to allow for the visualization and measurement of the total area of the bone defect. Subsequently, the external images of the bone defect were deleted, and only the images of the central portion of the defect were used for analysis (Figure 2).

Histomorphometric analysis consisted of the demarcation of the total area of the bone defect and of regenerated bone areas within the defect. These measurements were obtained in Pixel^2^ units using Axiovision^®^ software, version 4.6.3, (Carl Zeiss Imaging) and used to calculate the percentage area of the defect containing regenerated bone (Figure 2).

For statistical analysis of the bone fill percentage, we used the mixed models test with the covariance structure chosen by the smallest criterion using Akaike’s information, which, in this case, was the diagonal. The *post hoc* test used was Bonferroni’s test.

## RESULTS

The sample (N=90) did not show any postoperative infectious complications. However, one rat was excluded from the sample because of magnet displacement and another because the quality of the material obtained was unsuitable for histological assessment.

### Histomorphometric analysis

The bone fill percentage was calculated based on the total area of the bone defect and the size of the areas containing newly-formed bone in its interior. This variable showed a normal distribution. The model showed a time interaction of p=0.008. Mean values, standard errors, and confidence intervals for the bone fill percentages identified in each group are shown in Table 1.

**Table 1:** Bone fill percentages in all experimental groups after 15, 30, and 60 postoperative days

Group	Time (days)	Average	Standard error	Confidence interval of 95%	
				Lower limit	Upper limit
CT	15	1.631	1.643	-1.776	5.039
	30	8.335	3.413	1.29	15.38
	60	14.055	3.964	5.875	22.236
CT + M	15	3.989	1.47	0.941	7.037
	30	17.018	3.413	9.973	24.063
	60	19.12	3.964	10.940	27.300
HA	15	6.258	1.47	3.21	9.306
	30	19.372	3.413	12.327	26.417
	60	25.5	3.964	17.320	33.680
HA + M	15	3.811	1.47	0.764	6.859
	30	13.617	3.413	6.572	20.661
	60	31.199	3.964	23.019	39.380
AB	15	3.069	1.643	-0.339	6.476
	30	16.384	3.413	9.339	23.429
	60	15.544	3.964	7.364	23.724
AB + M	15	8.644	1.47	5.596	11.692
	30	29.041	3.413	21.996	36.086
	60	34.749	3.964	26.568	42.929

**Figure 3:**
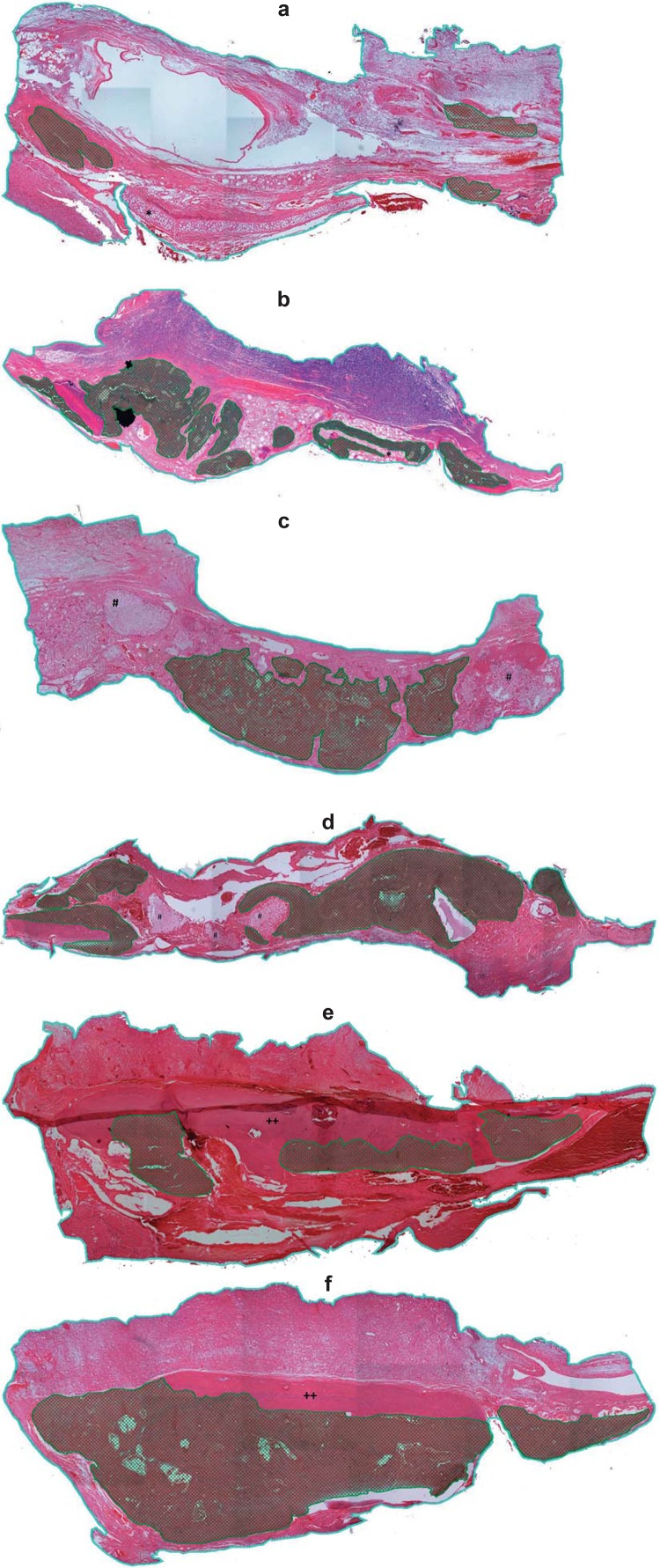
Histomorphometric analysis after reconstruction with autogenous bone graft, powdered synthetic hydroxyapatite implant, andallogeneic cartilage graft, with or without exposure to buried magnetic field, at 60 postoperative days (Blue lines define the total area of the bone defects, and cross hatched areas represent areas of new bone formation within the critical defect). d) Powdered synthetic hydroxyapatite group exposed to buried magnetic stimulation; e) Autogenous bone graft group not exposed to buried magnetic stimulation; f) Autogenous bone graft group exposed to buried magnetic stimulation. * indicates cartilage graft; # indicates hydroxyapatite implant; ++ indicates bone graft

Between-group comparisons of bone fill percentage at 15 postoperative days showed that the autogenous bone graft with magnetic field group (AB+M) had a higher bone fill percentage than the allogeneic cartilage graft without magnetic field group (CT) – indicated in the graph for *. At 30 postoperative days, the bone fill percentage of AB+M rats was still higher than observed in the CT group, and was also significantly greater than that seen in the hydroxyapatite implant with magnetic field group (HA+M) – indicated in the graph for #. After 60 days, the HA+M group showed a higher bone fill percentage than the CT group – indicated in the graph for +, and the AB+M group had higher bone fill than the CT and autogenous bone graft without magnetic field (AB) groups – indicated in the graph for **. These results showed that 60 days after autogenous bone reconstruction, rats exposed to magnetic fields had a higher bone fill percentage than those without stimulation (Figure 3).

**Figure 4:**
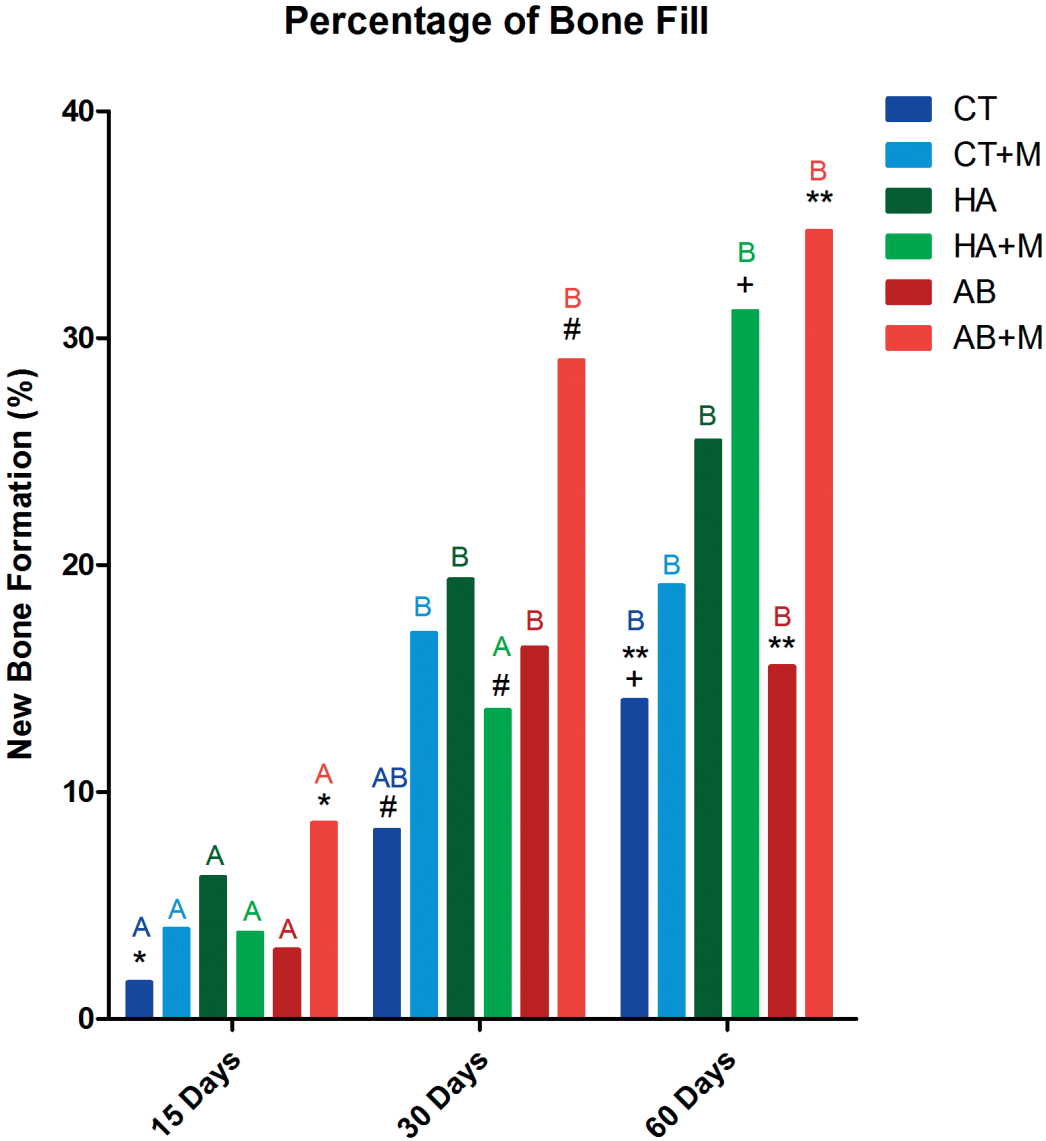
Graphical representation of the percentage of bone formation over time for all groups at 15 postoperative days: AB+M > CT (*); At 30 postoperative days: AB+M > CT (#); At 60 postoperative days: HA+M > CT (+); At 60 postoperative days: AB+M > CT e AB (**). Different capital letters show longitudinal differences

Longitudinal analyses were also performed to evaluate the development of each group over time. In the allogeneic cartilage graft with magnetic field (CT+M), hydroxyapatite implant without magnetic field (HA), AB, and AB+M groups, bone fill percentage after 15 days was lower than that found after 30 and 60 days. In HA+M rats, differences were only observed after 60 days, at which point bone fill percentages were significantly higher than those observed after 15 and 30 days. In the CT group, although differences in bone fill were observed between 15 and 60 days, bone fill at 30 postoperative days did not differ from that found after 15 and 60 days - represented in the graph by different capital letters. The graphical representation of the percentage of bone fill shows the between-group and longitudinal comparisons (Figure 4).

## DISCUSSION

The present study was based on the current understanding of the physiology of bone formation, and aimed to contribute to existing knowledge of the mechanisms involved in bone repair. The latter process is a key part of the response of the organism to bone tissue damage^13^ ^,^ ^23^.

In order to understand the influence of magnetic fields on bone repair, several studies have focused on histological parameters^20^ ^,^ ^21^, assessing the concentration of growth factors^1^ ^,^ ^11^, the deposition of calcium ions during ossification^26^, and even the influence of magnetism on cell plasma membranes^11^. The ability of different interventions to accelerate the bone repair process and contribute to the restoration of bone form and function has also been widely studied, often by the use of critical bone defects in rats^9^ ^,^ ^22^. Lastly, the quantification and comparison of tissues present in histological sections are generally performed by histomorphometric analysis^8^ ^,^ ^9^ ^,^ ^16^ ^,^ ^19^.

The present study evaluated the effects of a buried magnetic field on bone repair through the placement of magnets adjacent to a bone defect created according to the method outlined by Puricelli, et al.^21^ (2006). Our results revealed that, after 60 postoperative days, rats treated with autogenous bone reconstruction and exposed to magnetic fields showed significantly higher bone fill than those who received a similar treatment but no magnetic stimulation. In the early postoperative period, although the average fill percentage was higher in groups which received magnetic stimulation, these differences were not statistically significant.

Autogenous grafts are considered the gold-standard of bone-grafting, and in the present study, rats that received such a treatment in addition to magnetic stimulation had the highest percentage of bone fill. The osteoinductive, osteoconductive and osteogenetic properties of autogenous grafts are known to have a positive influence on bone repair. Our results also point to a sustained positive effect of magnetic field exposure on bone repair.

Although the present study has produced promising evidence of the influence of magnetic fields on bone repair, there is still a need to identify the best method to evaluate this process, and to clarify its underlying biological mechanisms. Studies on tissue engineering using magnetized scaffolds have shown promising results^4^, as well as the influence of the static magnetic field in cell culture^28^.

In addition to the potential positive effect of magnetic fields on bone graft healing, other factors may also have a direct influence on the incorporation of bone grafts. For instance, one factor is the type of graft used. The present study involved the use of block cortical grafts harvested from the cranial bone. This type of graft has shown slower revascularization than bone marrow grafts; consequently, the incorporation of the former is always slower than that of the latter^13^. Another factor which may influence graft healing is the size of the graft particles used. In a study conducted in rabbits, the early stages of bone repair were found to be influenced by the size of the autogenous bone particles in the grafts used^17^. In the present work, the use of block grafts probably had a negative influence on bone repair after 15 and 30 postoperative days. According to Shapoff, et al.^24^ (1980), the total volume of newly-formed bone in defects filled with small particles may be higher than that found in defects filled with larger particles after similar postoperative periods. Additionally, as Nagata, et al.^13^ (2009) have also pointed out, there is a need to establish a lower limit for particle size, since bone particles smaller than 125 µm are susceptible to removal by macrophages.

Despite being considered the gold standard for bone reconstruction, autogenous bone grafting is associated with several limitations, the most important of which are surgical morbidity at the donor site, the limited supply of graft quantity, and the irregular resorption of the graft^2^. Therefore, the present study sought to evaluate alternative materials that could effectively replace autogenous bone. The main requirements for the success of a bone substitute are biocompatibility, bioactivity, and adequate mechanical properties. Hydroxyapatite has been extensively studied as a bone substitute, and has been widely used for the treatment of bone defects. Its chemical formula is similar to that of inorganic bone tissue, which may explain its intense affinity to bone^7^. Although the quality of hydroxyapatite may vary between manufacturers, this has not had a significant impact on the results of studies involving the use of this material. However, the size of hydroxyapatite pores has been found to influence its filling by osteoblasts, and materials with a pore size of 150 to 500 µm are considered ideal for grafting^15^.

Our histological assessments allowed for the confirmation of the biocompatibility of the materials used, since graft rejection was not observed in any of the animals used. In the vast majority of cases, hydroxyapatite granules were surrounded by granulation tissue, suggesting possible future bone neoformation. The osteoconductivity of hydroxyapatite was also demonstrated in the present study, corroborating the results found by other authors^7^ ^,^ ^18^. Bone formation was observed on the surface of hydroxyapatite at all operative times.

The materials involved in the present study did not adapt easily to the contours of the bone defects, possibly because of the shallowness of the bone cavities themselves. This relative instability of the material can lead to variations in the amount of newly-formed bone within the same experimental group^7^. Some authors have used bone substitutes supported by membranes^9^ ^,^ ^10^ ^,^ ^12^ or to secure graft stability. The use of this method has led to promising results, especially when used in conjunction with growth factors and osteoinductive substances^7^ ^,^ ^14^.

The role of cartilage in bone formation and repair was also examined in the present study, since bone defects in one of the experimental groups were treated with allogeneic cartilage grafts. Cartilage grafts have been used by several authors in oral and maxillofacial reconstruction, and has been found to have several advantages associated with its long-term integrity and survival^5^. In the present study, the animals treated with allogeneic cartilage grafts showed the lowest percentage of bone fill, possibly because the resorption of this material was slower than that of the other bone substitutes used. In a study on rats performed by Vieira, et al.^25^ (1993), in which different means of cartilage graft preservation were compared, the authors found that the cartilage only began to be replaced by bone after 120 postoperative days.

The results obtained in the present study emphasize the importance of removing the bone segments between the magnets and the central region of the bone defect to allow for more intense magnetic fields and produce more favorable effects on bone repair.

## CONCLUSION

The present findings led to the following conclusions:

a) After 60 postoperative days, bone repair, as indicated by bone fill percentages, was exposed to a magnetic field and it was greater in the group treated with autogenous bone grafts than in the group treated with autogenous bone grafts and not exposed to magnetic fields;

b) Bone repair was most pronounced in animals treated with autogenous bone grafts, followed by those treated with powdered synthetic hydroxyapatite and allogeneic cartilage grafts.

The present research has contributed to the understanding of the influence of buried magnetic fields on bone repair. It is suggested that future studies invest in new methods that allow them to complement the present results and strengthen this line of research.
